# Supplementary figures and images for: Human RAP2A homolog of the Drosophila asymmetric cell division regulator Rap2l targets the stemness of glioblastoma stem cells
Source: eLife. 2025 Nov 28;14:RP105690. doi: 10.7554/eLife.105690 (PMC12662630; doi:10.7554/eLife.105690)

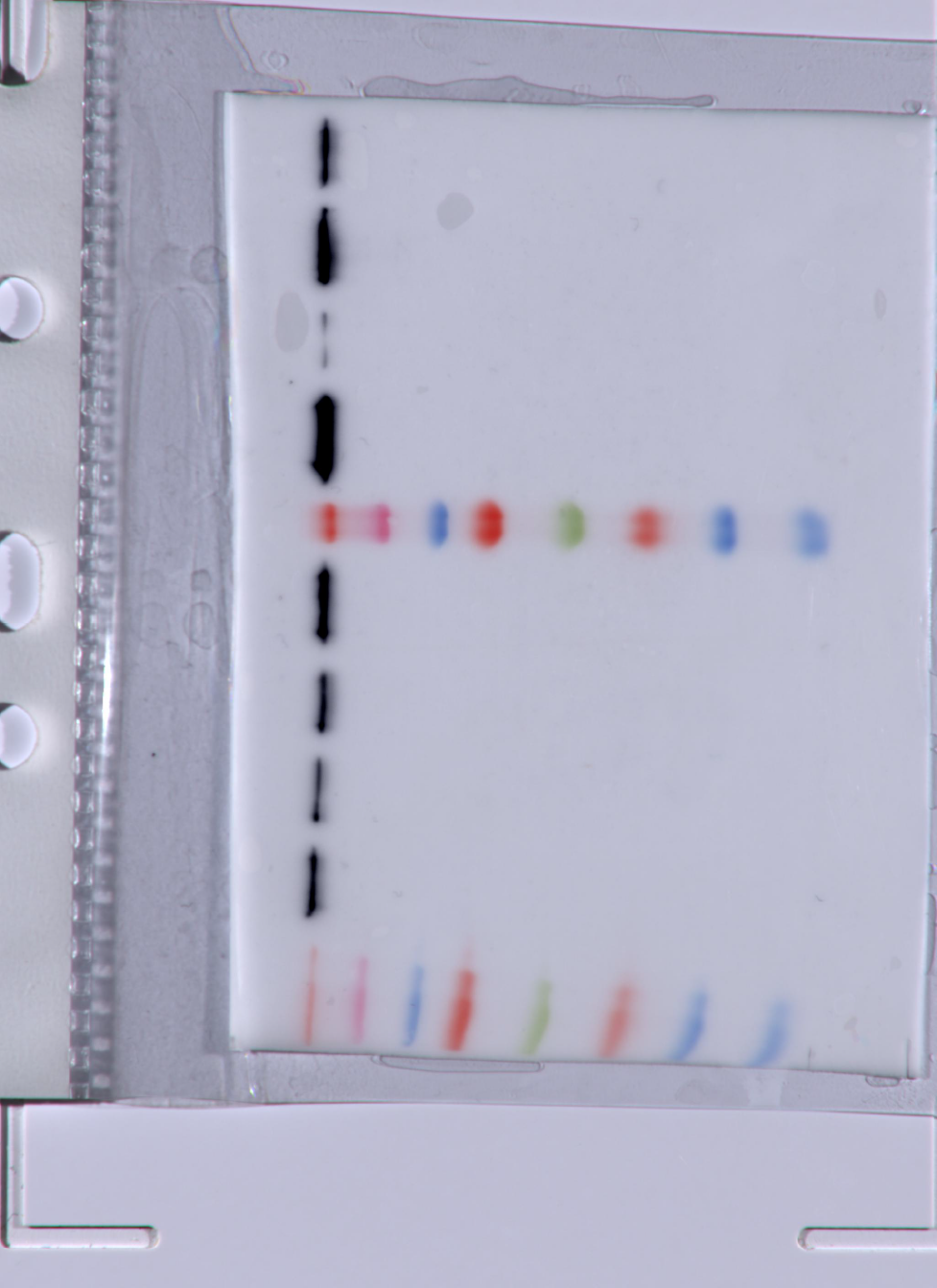

Supplement: Figure 4—source data 1. [file elife-105690-fig4-data1.zip › ORIGINAL BLOTS/18022021 MBC48 Nestin 2021.02.18_14.30.04_Ch+Marker.tif]

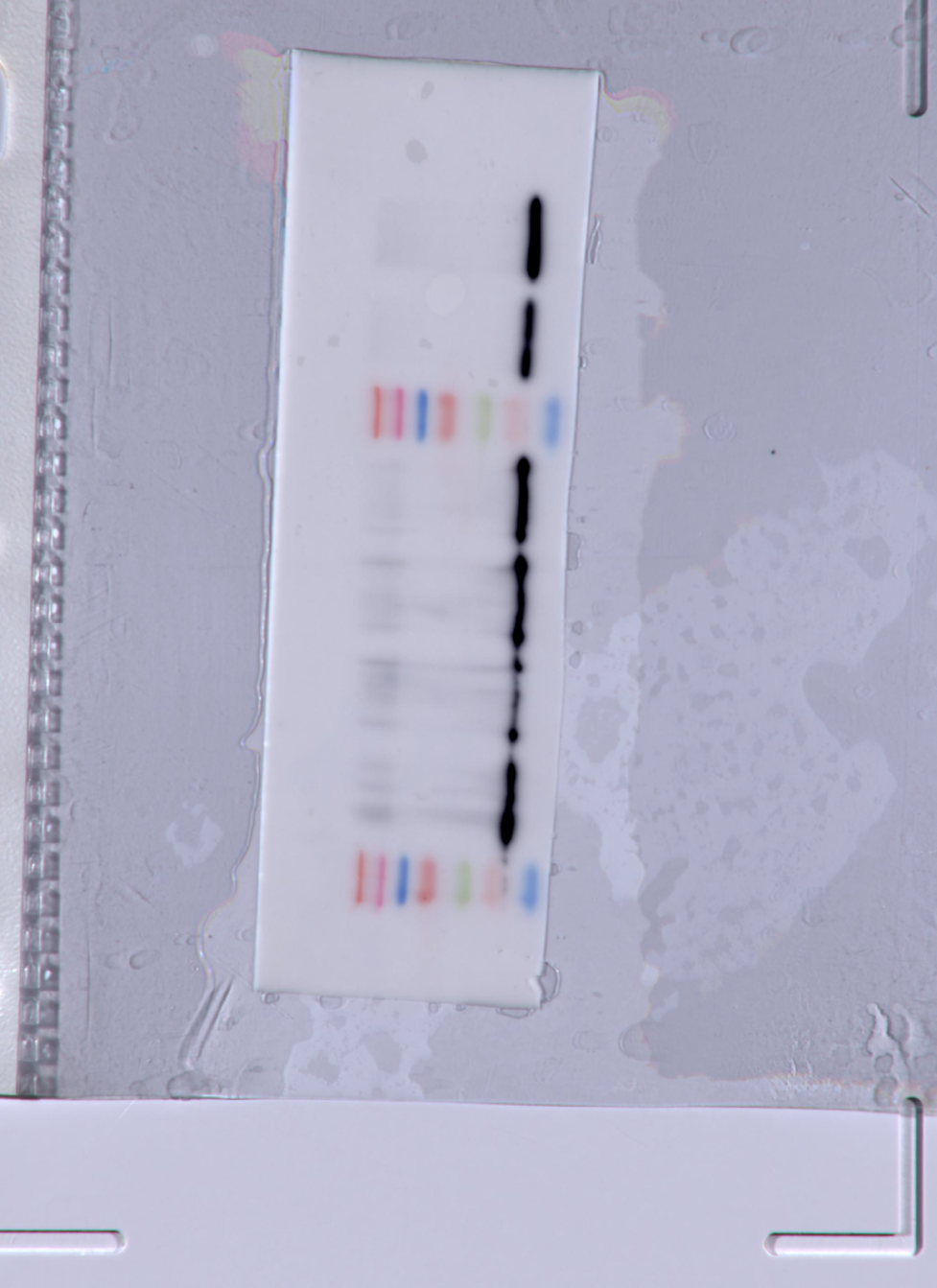

Supplement: Figure 4—source data 1. [file elife-105690-fig4-data1.zip › ORIGINAL BLOTS/GADPH MBC28A GADPH Auto 2020.02.25_16.38.32_Ch+Marker.tif]

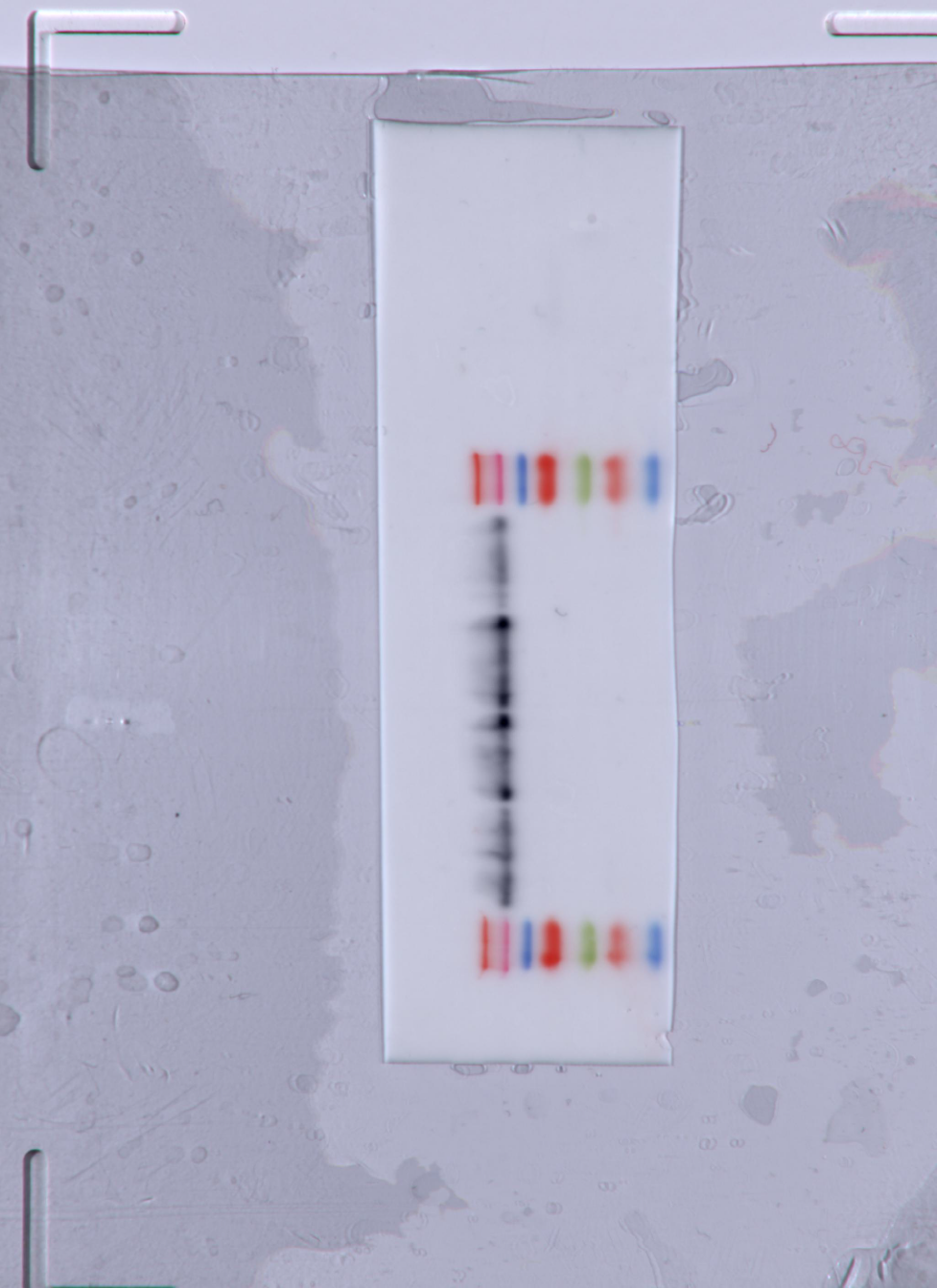

Supplement: Figure 4—source data 1. [file elife-105690-fig4-data1.zip › ORIGINAL BLOTS/MB28 20022020 CD133 cada 10 sec 2020.02.20_14.19.30-05_Ch+Marker (1).tif]

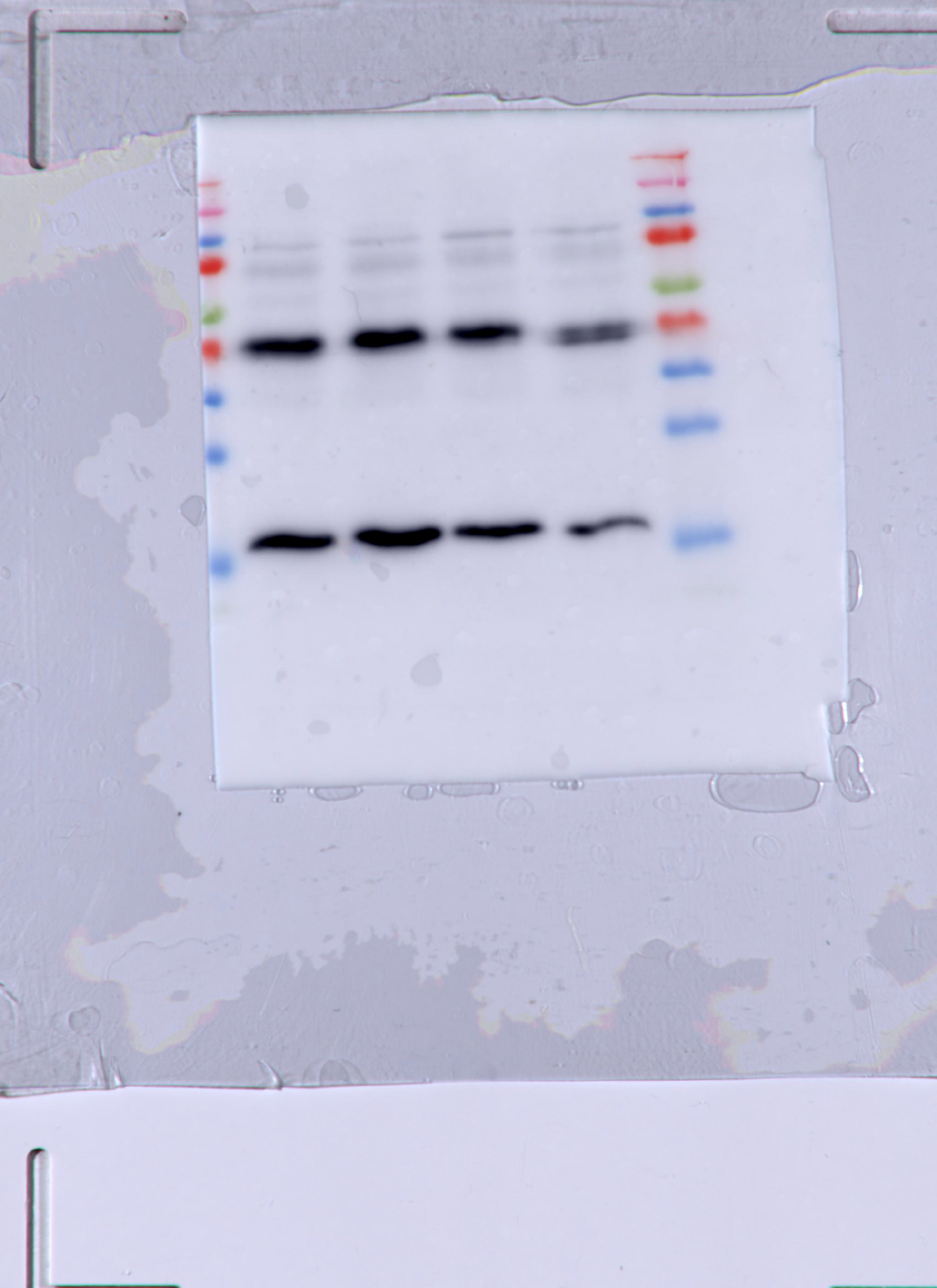

Supplement: Figure 4—source data 1. [file elife-105690-fig4-data1.zip › ORIGINAL BLOTS/MBC46 09022021 Sox auto 2021.02.09_14.36.07_Ch+Marker.tif]

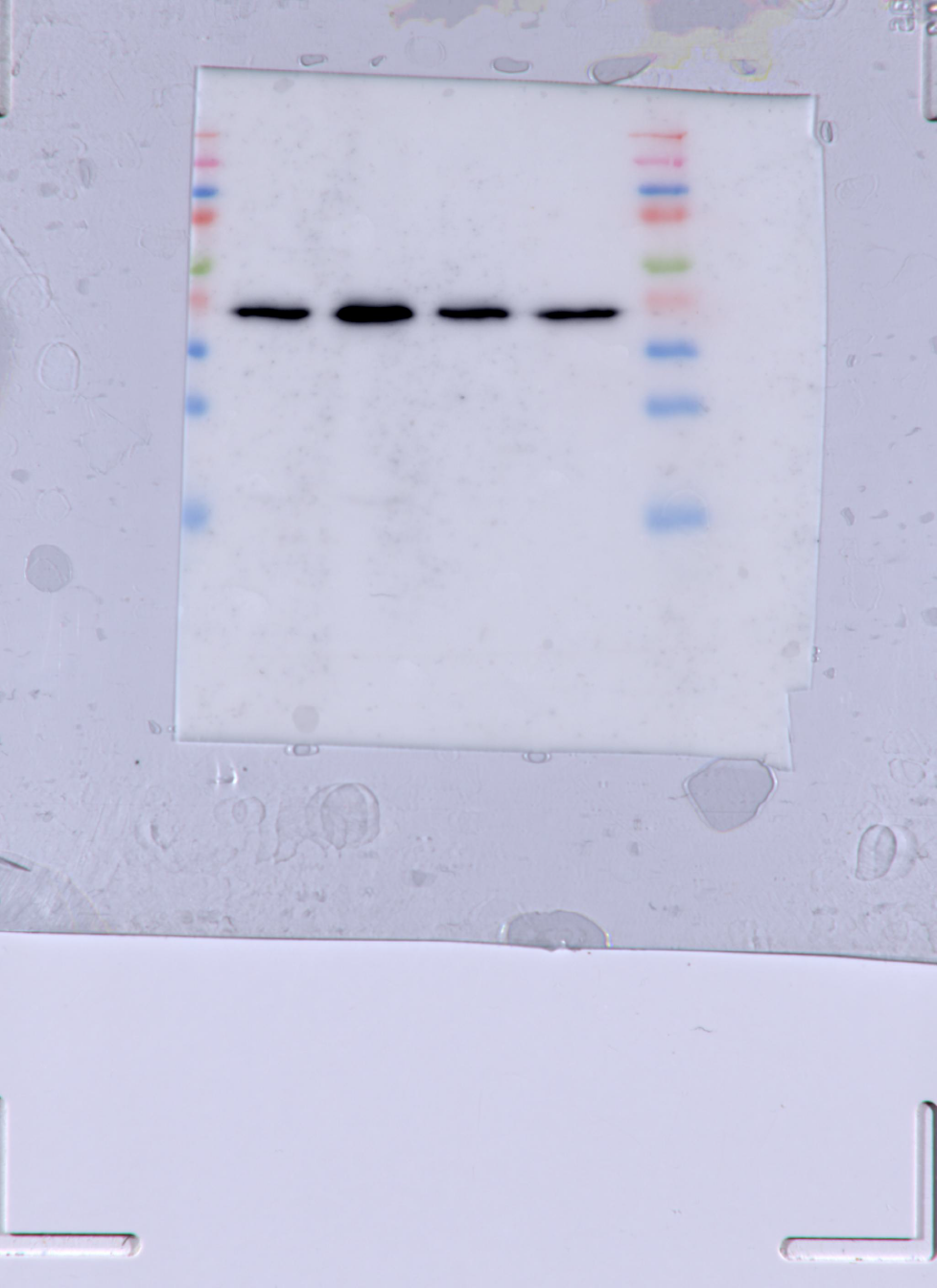

Supplement: Figure 4—source data 1. [file elife-105690-fig4-data1.zip › ORIGINAL BLOTS/MBC46B' 10022021 GADPH auto 2021.02.10_14.30.30_Ch+Marker.tif]

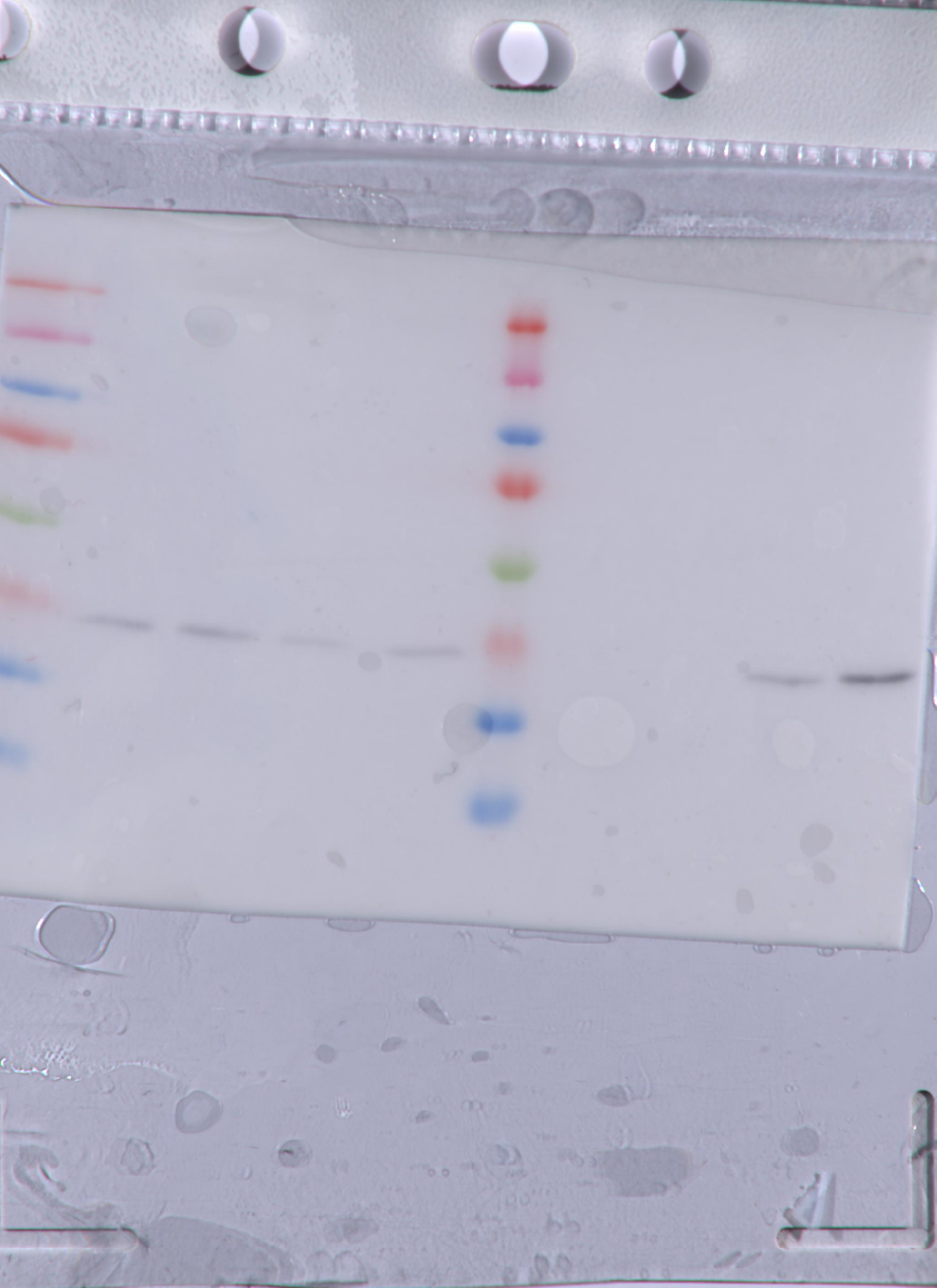

Supplement: Figure 4—source data 1. [file elife-105690-fig4-data1.zip › ORIGINAL BLOTS/MBC48 23022021 GADPH 2021.02.23_15.54.51-05_Ch+Marker.tif]

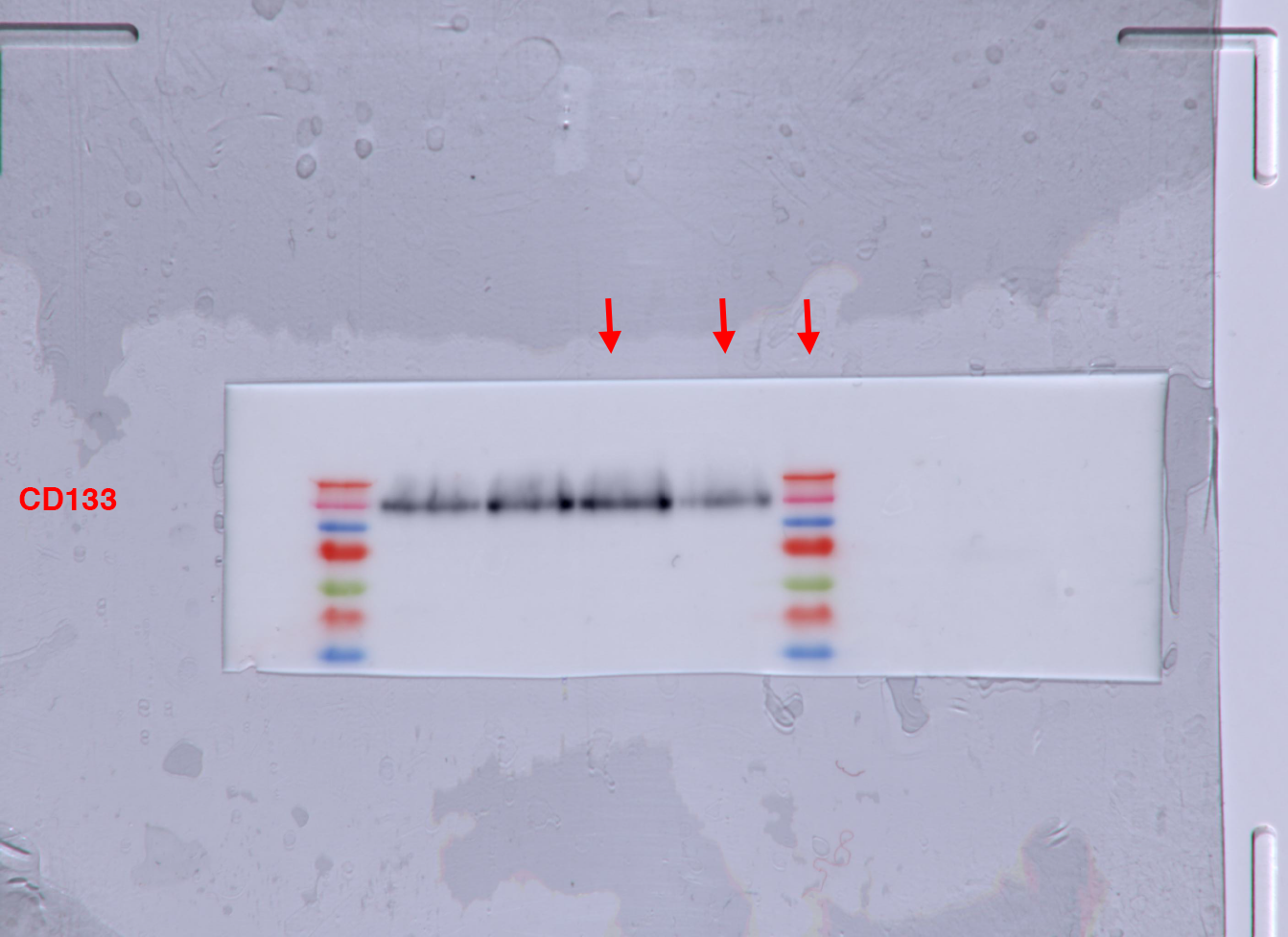

Supplement: Figure 4—source data 2. [file elife-105690-fig4-data2.zip › 1. CD133 Originals with added arrows (to indicate the lanes shown in the figure4) copy/1.1. MB28 20022020 CD133 cada 10 sec 2020.02.20_14.19.30-05_Ch+Marker (1).tif]

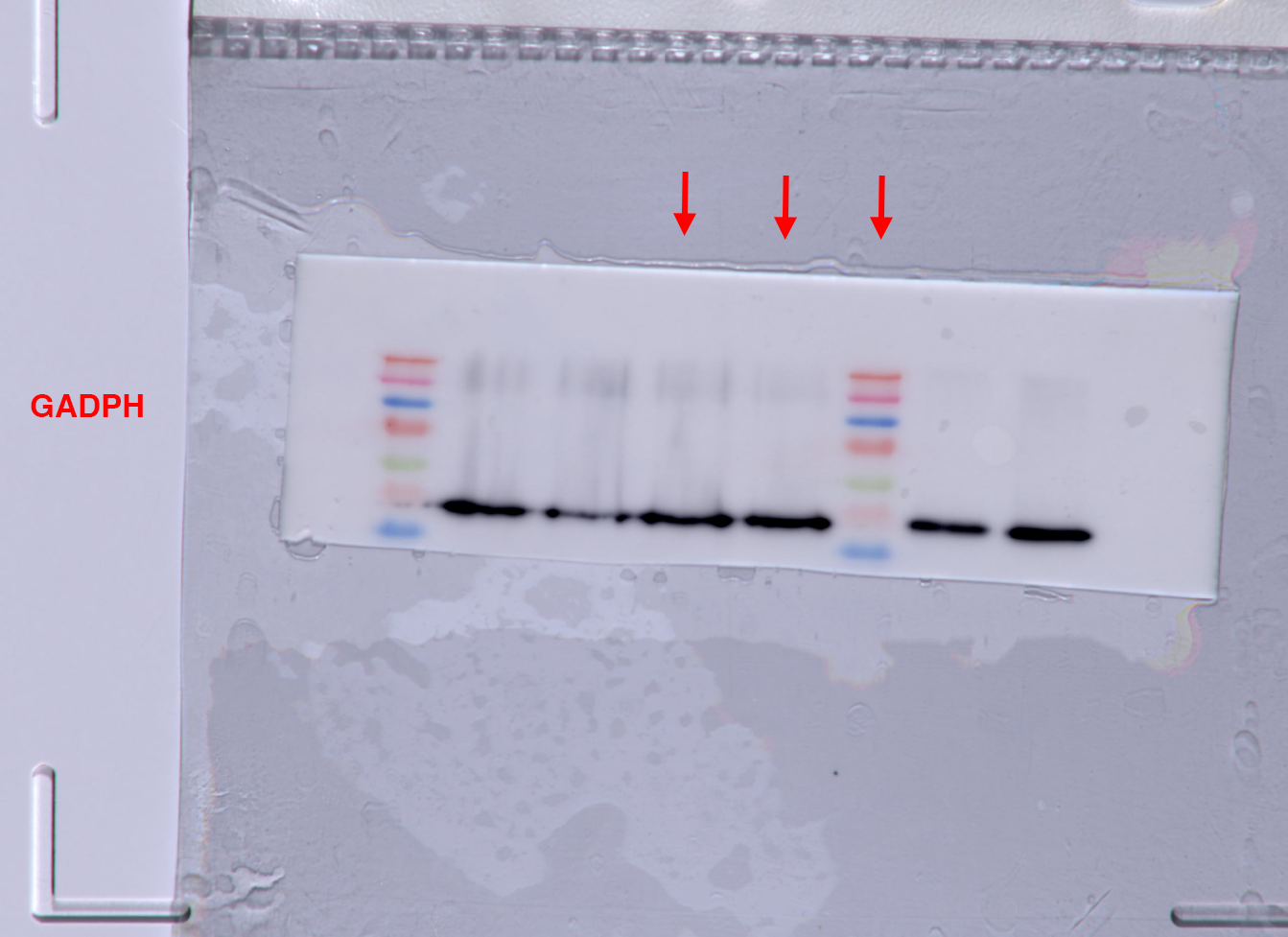

Supplement: Figure 4—source data 2. [file elife-105690-fig4-data2.zip › 1. CD133 Originals with added arrows (to indicate the lanes shown in the figure4) copy/1.2.GADPH MBC28A GADPH Auto 2020.02.25_16.38.32_Ch+Marker.tif]

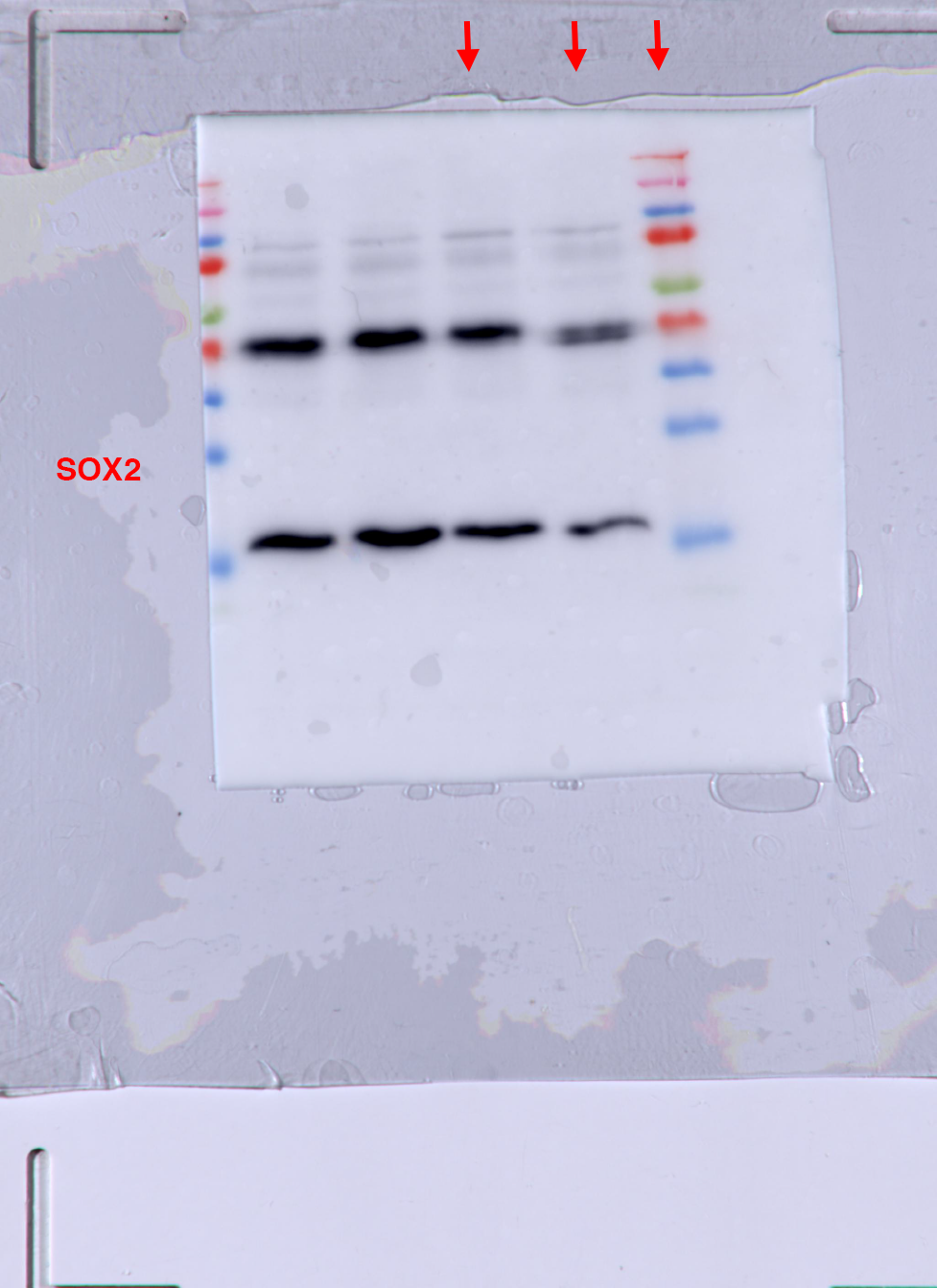

Supplement: Figure 4—source data 2. [file elife-105690-fig4-data2.zip › 2. SOX2 Originals with added arrows (to indicate the lanes shown in the figure4) copy/2.1. MBC46 09022021 Sox auto 2021.02.09_14.36.07_Ch+Marker.tif]

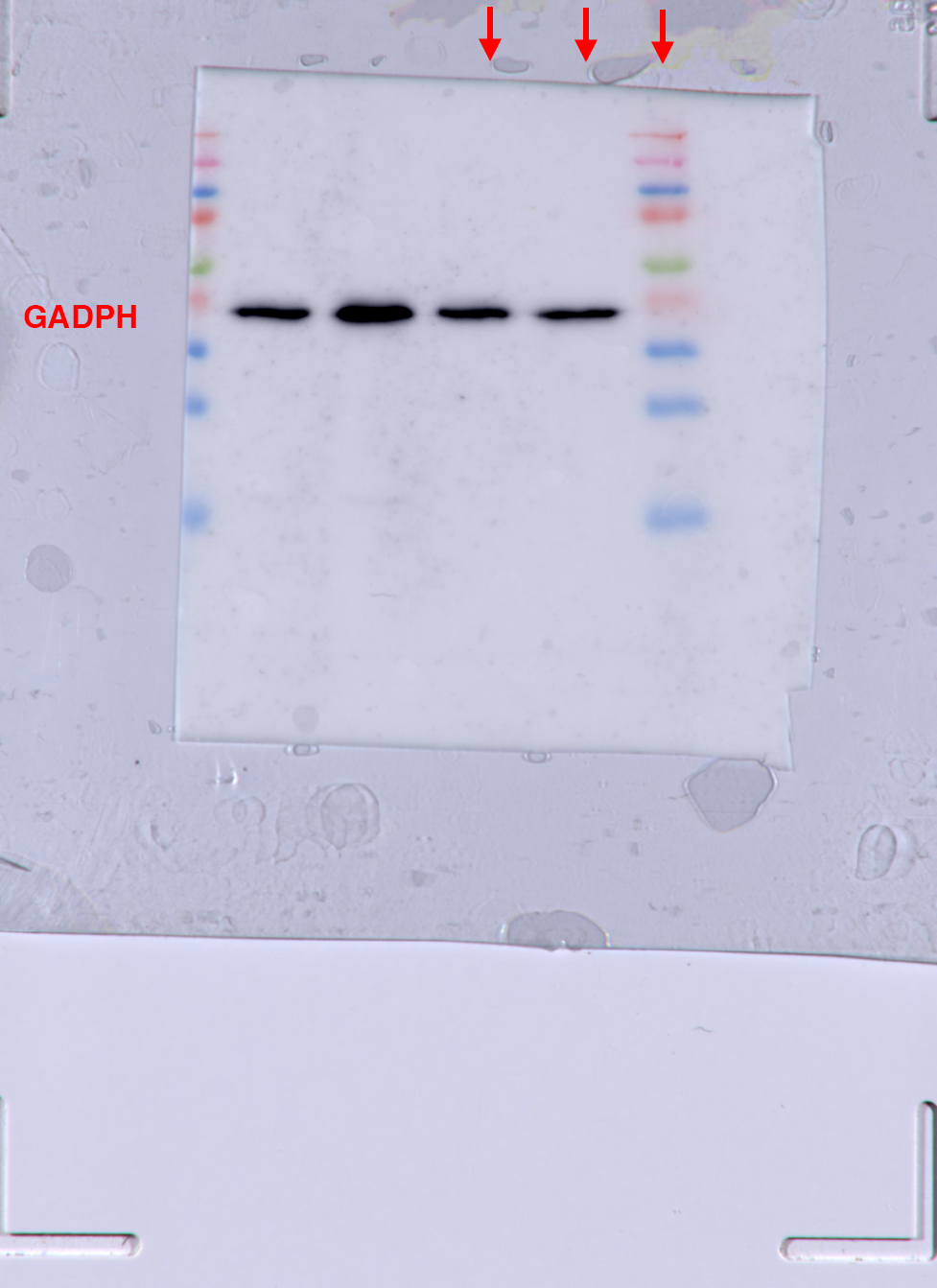

Supplement: Figure 4—source data 2. [file elife-105690-fig4-data2.zip › 2. SOX2 Originals with added arrows (to indicate the lanes shown in the figure4) copy/2.2. MBC46B' 10022021 GADPH auto 2021.02.10_14.30.30_Ch+Marker.tif]

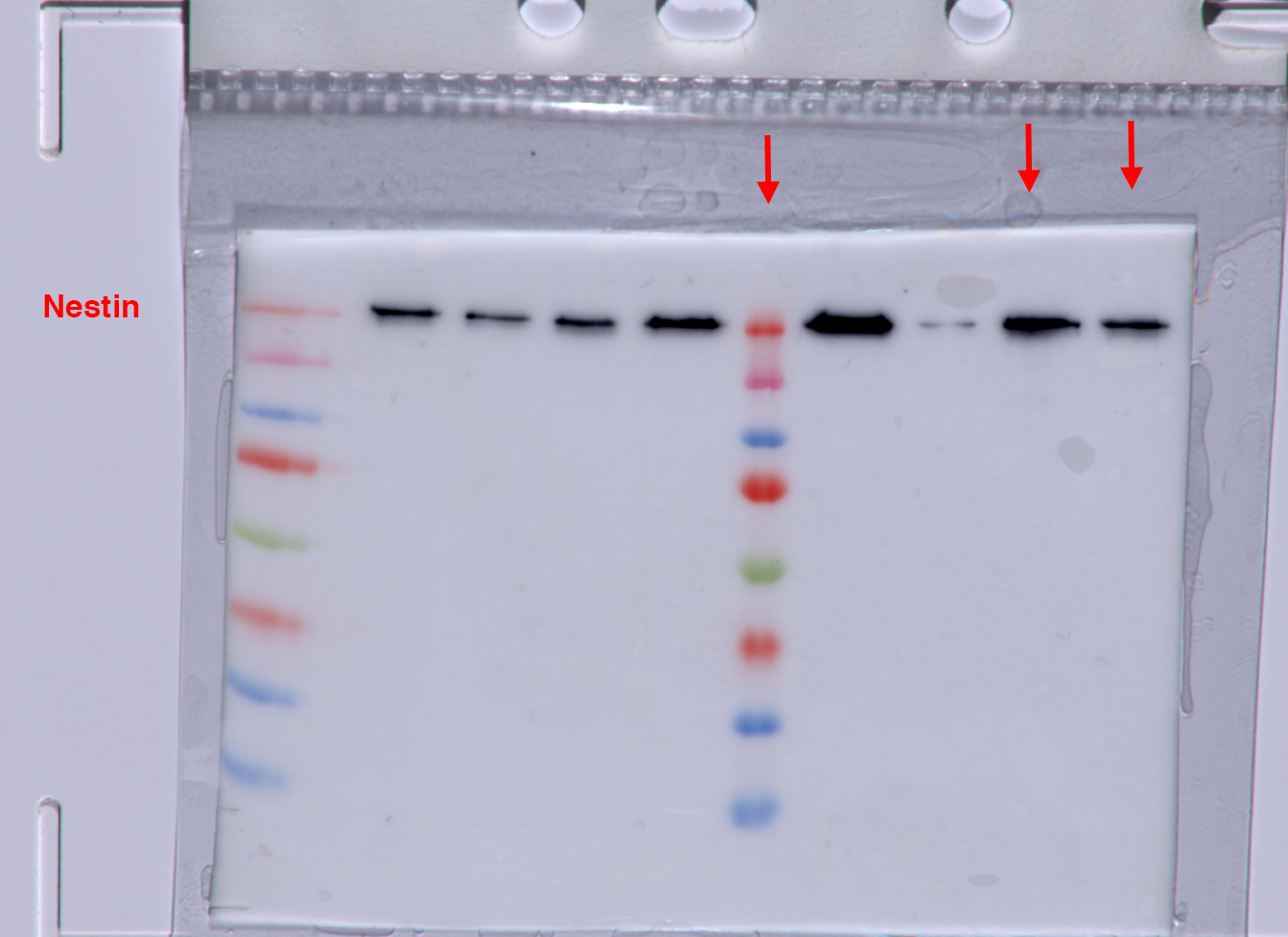

Supplement: Figure 4—source data 2. [file elife-105690-fig4-data2.zip › 3. Nestin Originals with added arrows (to indicate the lanes shown in the figure4) copy/3.1. 18022021 MBC48 Nestin 2021.02.18_14.30.04_Ch+Marker.tif]

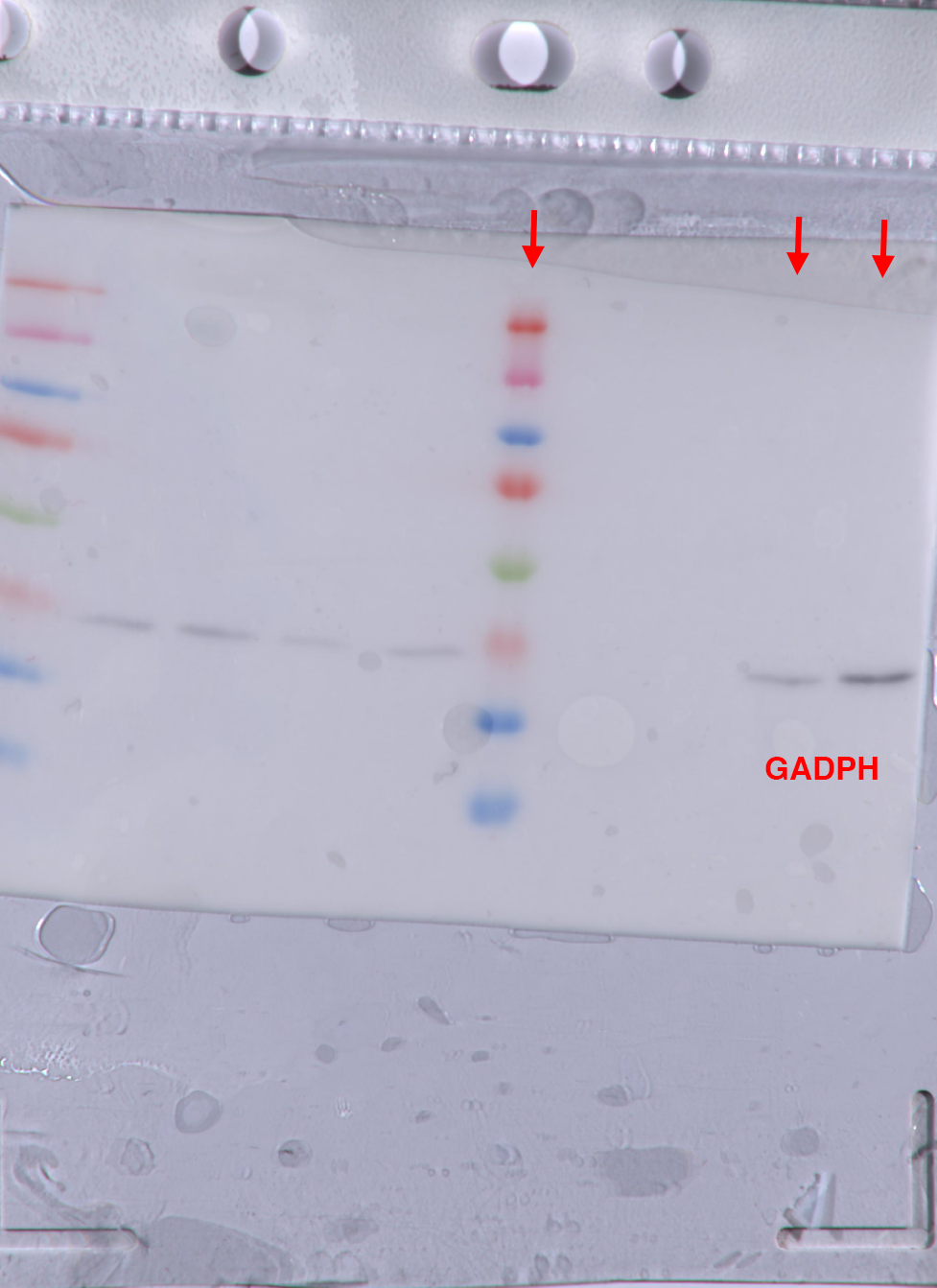

Supplement: Figure 4—source data 2. [file elife-105690-fig4-data2.zip › 3. Nestin Originals with added arrows (to indicate the lanes shown in the figure4) copy/3.2. MBC48 23022021 GADPH 2021.02.23_15.54.51-05_Ch+Marker.tif]
